# Tumor-responsive PEGylated mesoporous nanoparticles achieve enhanced chemotherapy and reduced toxicity in prostate cancer

**DOI:** 10.1016/j.ijpx.2026.100492

**Published:** 2026-01-27

**Authors:** Yangyang Song, Xue Tan, Kai Yu, Yue Wang, Jixue Wang

**Affiliations:** a Department of Urology, the First Hospital of Jilin University, Changchun 130021, PR China; b Department of Nursing Platform for Breast Surgery, the First Hospital of Jilin University, Changchun 130021, PR China; c Department of Operation Room, the First Hospital of Jilin University, Changchun 130021, PR China

**Keywords:** PEGylated mesoporous silica nanoparticles, Redox-responsive drug delivery, Disulfide-bridged nanocarriers, Docetaxel chemotherapy, Prostate cancer therapy

## Abstract

Docetaxel (DTX) remains the first-line chemotherapeutic for advanced prostate cancer, however, its therapeutic efficacy remains limited by poor aqueous solubility, rapid systemic clearance, and severe dose-dependent toxicity. To overcome these constraints, we developed a PEGylated, disulfide-bridged hierarchical mesoporous silica nanocarrier (PEG–HMS) as a redox-sensitive delivery system for DTX (PEG–HMS–DTX). The nanostructure was fabricated by integrating disulfide-containing organosilanes into the silica framework and conjugating thiol-reactive PEG chains, thereby combining long circulation stability with tumor-selective release. Comprehensive physicochemical characterization confirmed uniform spherical morphology, an optimal hydrodynamic size (∼40–50 nm), attenuated surface charge following PEGylation, and high colloidal stability in physiological media, while disulfide linkages enabled responsive structural changes under reductive conditions. Drug release was minimal under physiological conditions (<30% at 72 h) but markedly accelerated in the presence of glutathione (∼60% at 72 h). Compared with free DTX or non-PEGylated carriers, PEG-HMS-DTX exhibited stronger cellular uptake and enhanced cytotoxicity in RM-1 prostate cancer cells. In tumor-bearing mice, PEG-HMS-DTX achieved superior tumor accumulation (peak at ∼12 h), pronounced tumor growth inhibition (>70%), minimal systemic toxicity, and elevated apoptosis characterized by increased cleaved caspase-3 and reduced PCNA/Bcl-2 expression. Collectively, this “stable-in-circulation, trigger-in-tumor” platform substantially improves intratumoral DTX delivery and apoptosis-driven antitumor efficacy, while maintaining systemic safety. These findings highlight PEG-HMS-DTX as a promising and generalizable strategy for prostate cancer chemotherapy, warranting further pharmacokinetic, immunogenicity, and GLP toxicology studies to support translational advancement.

## Graphical abstract

A tumor-responsive PEGylated mesoporous silica nanoplatform (PEG–HMS–DTX) integrates a stealth PEG corona with an internally disulfide-crosslinked structure to achieve “stable in circulation, trigger in tumor” delivery of docetaxel. The nanosystem exhibits prolonged systemic persistence, preferentially accumulates at prostate tumor sites, and undergoes glutathione-mediated disassembly in the reductive tumor microenvironment, resulting in on-site drug release. This mechanism enhances apoptotic killing of cancer cells, markedly inhibits tumor growth, and reduces off-target toxicity compared with free docetaxel.

**Figure:**
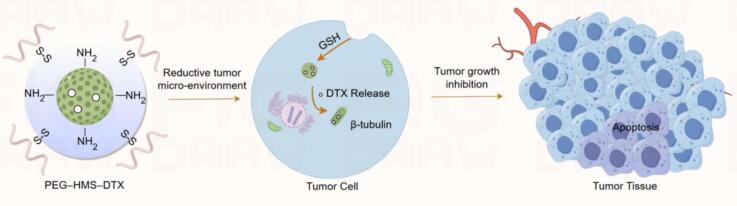

## 1 Introduction

Prostate cancer continues to rank among the most common malignancies in men globally and represents a major contributor to cancer-associated morbidity and mortality (Raychaudhuri et al., 2025; Ye et al., 2022). For advanced and metastatic disease, taxane-based chemotherapy—most notably docetaxel (DTX)—constitutes a cornerstone of systemic treatment, improving survival and palliation in many patients (Iannelli et al., 2020; Mediavilla-Varela et al., 2009). Nevertheless, the clinical utility of DTX is constrained by several well-recognized limitations, including poor aqueous solubility, rapid systemic clearance, off-target distribution that produces dose-limiting toxicities (*e.g.*, myelosuppression, hepatotoxicity, nephrotoxicity), as well as the progressive development of drug resistance (Ganju et al., 2014; Sekino and Teishima, 2020). These shortcomings motivate continued efforts to develop delivery strategies that increase intratumoral drug exposure while minimizing systemic side effects.

Nanoparticle-mediated drug delivery has shown great potential in addressing these challenges by improving solubility, prolonging circulation time, and enhancing tumor selectivity *via* passive and active targeting mechanisms (Dang and Guan, 2020; Singh and Lillard Jr, 2009; Singh et al., 2011). Among inorganic nanocarriers, hollow mesoporous silica (HMS) nanoparticles are of particular interest due to their extensive surface area, adjustable pore architecture, high flexibility for surface engineering, and robust physicochemical stability (Wu et al., 2011; Wu et al., 2013). HMS can achieve high drug loading, protect payloads from premature degradation, and be engineered for stimuli-responsive release. However, translation of HMS has been limited by two interrelated issues: premature drug leakage during circulation and rapid elimination through the mononuclear phagocyte system (MPS) (Yousefiasl et al., 2025). Surface modification and responsive frameworks have therefore become central strategies to reconcile systemic stability with efficient intratumoral drug delivery (Abdo et al., 2020; Kankala et al., 2020; Rahikkala et al., 2018; Zhang et al., 2015).

Polyethylene glycol (PEG) grafting (PEGylation) is widely used to impart “stealth” characteristics—reducing protein adsorption and MPS uptake and thereby extending systemic half-life (Cauda et al., 2010; Li et al., 2020). Yet, excessive or improperly configured PEG layers can impede cellular uptake and limit intracellular drug release, reducing therapeutic potency. Complementarily, introduction of labile chemical linkages into the carrier matrix—such as disulfide bonds that are cleavable under intracellular reductive conditions—provides a tumor-selective trigger for drug release, exploiting the substantially higher glutathione (GSH) concentrations found inside many tumor cells relative to plasma (Gong et al., 2015). Integrating PEGylation with an internally disulfide-bridged HMS architecture offers a rational route to achieve the desirable “stable in circulation — trigger in tumor” behavior, but doing so requires careful control of PEG density, chain length, and the placement of redox-responsive motifs to preserve both prolonged circulation and effective cellular delivery (Cui et al., 2012; Wang et al., 2015; Xie et al., 2016).

In this work, we constructed a PEGylated, disulfide-bridged hierarchical mesoporous silica nanoplatform (PEG-HMS) for delivery of docetaxel (PEG-HMS-DTX). Our design couples an internal disulfide-containing organosilane backbone with surface thiol chemistry that enables reversible PEG anchoring, creating a carrier that remains colloidally stable under physiological conditions yet undergoes controlled disassembly/release in reductive tumor microenvironments. We hypothesized that this dual-feature architecture would (i) reduce premature systemic drug release and MPS clearance *via* PEG shielding, (ii) enhance passive tumor localization by particle size optimization and exploitation of the enhanced permeability and retention (EPR) effect, and (iii) enable efficient intracellular DTX liberation *via* GSH-mediated disulfide cleavage, thereby enhancing apoptosis induction and antitumor efficacy while reducing off-target toxicity.

To validate these hypotheses, we performed systematic physicochemical analyses, *in vitro* intracellular accumulation and cytotoxicity assays, *in vitro* redox-responsive release studies, and *in vivo* evaluations including tumor biodistribution, antitumor efficacy in an RM-1 prostate cancer model, and systemic biosafety assessment. The results provide mechanistic insight into how PEG shielding and internal disulfide bridges cooperatively shape pharmacokinetics, tumor selectivity, and downstream apoptotic responses (*e.g.*, modulation of Bcl-2 and caspase-3). Importantly, our data demonstrate that the integrated PEG-HMS-DTX platform achieves a favorable therapeutic index relative to free DTX and non-PEGylated counterparts.

While promising, several translational challenges remain—most notably the heterogeneity of EPR in clinical tumors, potential anti-PEG immune responses, and the need for rigorous long-term toxicology and GLP-level pharmacokinetic studies (Hussain et al., 2019; Rosenholm et al., 2010; Yousefiasl et al., 2025). Nevertheless, by combining steric stabilization with an intrinsic tumor-triggered release mechanism, PEG-HMS-DTX represents a practical and generalizable strategy to improve chemotherapy delivery for prostate cancer and potentially other solid tumors characterized by elevated intracellular reducing conditions. The following sections describe the preparation, physicochemical analysis, and bioassessment of this system and discuss its implications for future preclinical development and clinical translation.

## 2 Materials and methods

### 2.1 Materials

Docetaxel (DTX, ≥99% purity) was obtained from Aladdin Biochemical Technology Co., Ltd. (Shanghai, China). Cetyltrimethylammonium bromide (CTAB), tetraethyl orthosilicate (TEOS), ammonium nitrate, sodium hydroxide (NaOH), ethanol (96%), hydrochloric acid (HCl), and ammonium hydroxide were supplied by Sinopharm Chemical Reagent Co., Ltd. (Shanghai, China). (3-Aminopropyl)triethoxysilane (APTES), bis[3-(triethoxysilyl)propyl] disulfide (BTESPD), and 3-mercaptopropyl trimethoxysilane were purchased from Sigma-Aldrich (St. Louis, MO, USA). Methoxy poly(ethylene glycol) thiol (mPEG–SH, MW 2000 Da) was acquired from JenKem Technology Co., Ltd. (Beijing, China). Fluorescein isothiocyanate (FITC) was obtained from Beyotime Biotechnology (Shanghai, China). Dulbecco's Modified Eagle Medium (DMEM, high glucose) and fetal bovine serum (FBS) were supplied by Gibco (Thermo Fisher Scientific, Waltham, MA, USA). Penicillin–streptomycin solution was obtained from Solarbio Life Sciences (Beijing, China). All other reagents and solvents were of analytical grade and used as received without additional purification. Ultrapure water was produced using a Milli-Q purification system (Merck Millipore, Darmstadt, Germany).

### 2.2 Synthesis of Hollow Mesoporous Silica (HMS) nanoparticles

Hollow mesoporous silica (HMS) nanoparticles were prepared through a modified sol–gel approach employing cetyltrimethylammonium bromide (CTAB) acting as a soft mold. The synthesis followed previously established protocols with slight modifications (Shahbaz et al., 2024; Zhu et al., 2011). In a typical procedure, CTAB (1.0 g) and sodium hydroxide (280 mg) were completely dispersed in 400 mL of deionized water with continuous stirring at 80 °C for 15 min. Subsequently, tetraethyl orthosilicate (TEOS, 5.36 mL) was slowly introduced dropwise, leading to the gradual formation of an opalescent suspension that indicated silica micelle generation. The reaction was maintained under these conditions for an additional 2 h to facilitate the growth of the mesoporous silica framework.

The resulting colloidal suspension was cooled and subjected to centrifugation (20,000 rpm, 4 °C, 15 min). The precipitate was washed repeatedly (three cycles) with 50% ethanol to remove loosely bound surfactant. Template extraction was subsequently conducted in two sequential reflux steps: first, the precipitate was redispersed in 100 mL of 96% ethanol containing 520 mg of ammonium nitrate and refluxed overnight; second, the recovered particles were refluxed again in 100 mL of 96% ethanol with 5 mL of concentrated aqueous hydrochloric acid overnight. After each extraction, the solids were collected *via* centrifugation, thoroughly rinsed with ethanol, and finally lyophilized to yield dry, uniform HMS nanoparticles.

### 2.3 Amination of HMS (HMS-NH_2_) and preparation of Docetaxel (DTX)-loaded nanoparticles

Approximately 700 mg of the as-prepared HMS powder was ultrasonically dissolved in 80 mL of anhydrous toluene. After the dispersion became homogeneous, 1.5 mL of (3-aminopropyl)triethoxysilane (APTES) was introduced dropwise while maintaining continuous magnetic stirring. The reaction proceeded under reflux for 24 h, enabling covalent attachment of amino functionalities onto the silica surface through silane condensation. The obtained HMS–NH₂ particles were recovered by centrifugation (17,000 rpm, 4 °C, 15 min), thoroughly rinsed with 96% ethanol several times, and vacuum-dried to remove residual solvent.

For DTX loading, 30 mg of HMS–NH₂ was suspended in 5 mL of a docetaxel solution (0.3 mg·mL^−1^ in PBS) and stirred under light-protected conditions for 24 h to ensure complete adsorption of the drug. Then, 10 mL of deionized water was slowly added while stirring to facilitate equilibrium redistribution, after which the suspension was subjected to dialysis against deionized water for 24 h to eliminate free DTX and residual impurities. The purified HMS–DTX nanoparticles were lyophilized and stored at 4 °C in the dark.

For encapsulation analysis, 1 mL of the HMS–DTX suspension (1 mg·mL^−1^) was placed in an ultrafiltration tube and centrifuged at 4000 rpm for 20 min. The retained nanoparticles were resuspended in deionized water, while the filtrate was mixed with acetonitrile (20 μL sample + 180 μL solvent) to dissociate micellar structures before quantification. The concentration of DTX was measured using high-performance liquid chromatography (HPLC), and the peak areas corresponding to free and total drug contents were denoted as A₁ and A₂, respectively. The values of drug loading content (DLC) and drug loading efficiency (DLE) were obtained by the corresponding calculation (1), (2):

(1) $$\mathrm{DLC}\;\left(\%\right)=\frac{\text{Amount of drug in nanoparticle}\;}{\text{Amount of loading nanoparticle}\;}\times 100\%$$

(2) $$\mathrm{DLE}\;\left(\%\right)=\frac{\text{Amount of drug in nanoparticle}\;}{\text{Total amount of feeding drug}}\times 100\%$$

### 2.4 Synthesis of PEGylated Hollow Mesoporous Silica (PEG-HMS) nanoparticles

PEGylated disulfide-bridged hollow mesoporous silica (PEG–HMS) nanoparticles were fabricated through a sequential surface modification strategy involving amine functionalization, incorporation of disulfide linkages, and PEG conjugation. To introduce the redox-responsive disulfide framework, 200 mg of HMS–NH₂ was ultrasonically suspended in 27 mL of deionized water containing 3 mL of 96% ethanol under vigorous stirring. A precursor solution consisting of 113 μL of bis[3-(triethoxysilyl)propyl] disulfide (BTESPD) and 226 μL of tetraethyl orthosilicate (TEOS) was then introduced dropwise. Afterward, an NaOH solution (5 mL, 4.8 mg·mL^−1^) was introduced into initiate the sol–gel condensation. The reaction was kept at room temperature for 30 min, forming disulfide-bridged HMS (HMS–NH₂–SiSS). The obtained particles were isolated through centrifugation, completely rinsed with 50% ethanol, and dried *in vacuo*. For subsequent thiol modification, 100 mg of HMS–NH₂–SiSS was redispersed in 15 mL of anhydrous toluene, then gradually adding 200 μL of 3-mercaptopropyl trimethoxysilane (MPTMS). The mixture was refluxed overnight under nitrogen protection to introduce surface –SH groups, generating thiolated HMS (HMS–NH₂–SiSS–SH). Finally, 50 mg of the thiolated nanoparticles were suspended in ethanol containing methoxy poly(ethylene glycol) thiol (mPEG–SH), and the dispersion was gently purged with oxygen for 2 h to promote the generation of disulfide linkages between mPEG–SH and surface thiols. The resulting PEGylated nanoparticles (PEG–HMS) were collected by centrifugation, washed repeatedly with ethanol, and vacuum-dried for subsequent use.

### 2.5 Characterization of nanoparticles

The structural and textural features of the prepared nanoparticles were thoroughly analyzed by a range of physicochemical techniques. The crystalline phase composition was examined by wide-angle X-ray diffraction (XRD) on a Bruker D8 Advance diffractometer equipped with Cu Kα radiation (λ = 1.5406 Å, 40 kV, 40 mA). Data were recorded over a 2θ range of 10°–80° with an incremental step of 0.02°. The ordered mesostructure was further assessed by small-angle X-ray diffraction (SAXRD) recorded in the 2θ range of 0.5°–10°, providing insight into the periodic pore arrangement within the HMS framework.

Fourier-transform infrared (FTIR) spectroscopy was conducted on a Nicolet iS50 instrument in the wavenumber region of 4000–400 cm^−1^ using KBr pellets to verify surface functional groups and chemical bonding characteristics. Nitrogen adsorption–desorption measurements were conducted on a Micromeritics ASAP 2460 analyzer at 77 K after degassing the samples at 120 °C for 12 h. The Brunauer–Emmett–Teller (BET) model was applied to determine the specific surface area, while pore size distribution was evaluated according to the Barrett–Joyner–Halenda (BJH) approach based on the desorption isotherm.

The morphology, particle size, and zeta potential of HMS–DTX and PEG–HMS–DTX nanoparticles were analyzed by transmission electron microscopy (TEM) and dynamic light scattering (DLS). For TEM observation, the nanoparticle suspension (0.1 mg·mL^−1^ in deionized water) was gently ultrasonicated to ensure uniform dispersion. A 10 μL aliquot was then deposited onto a carbon-coated copper grid and air-dried at room temperature, and stained with 1% phosphotungstic acid for approximately 1 min. After gently removing the residual stain using filter paper, the specimens were examined under TEM to visualize morphology and internal structure. For DLS measurements, 6.0 mL of nanoparticle dispersion (0.1 mg·mL^−1^) was analyzed to determine hydrodynamic diameter, polydispersity index (PDI), and ζ-potential using a Malvern Zetasizer (Malvern Instruments, UK) following sonication in deionized water. The colloidal stability and redox-responsiveness of the nanocarriers were further evaluated by monitoring size variations in phosphate-buffered saline (PBS) and fetal bovine serum (FBS) at various time points (0, 4, 8, 12, 24, 48, and 72 h). To assess redox sensitivity, 10 mM glutathione (GSH) was introduced into the system, and particle size evolution was recorded over the same time course.

### 2.6 *In vitro* redox-responsive DTX release

The *in vitro* release profile of DTX from different nanocarrier formulations was explored in phosphate-buffered saline (PBS, pH 7.4) under both reductive and non-reductive environments using a dialysis approach. In a standard experiment, 1.0 mg of HMS-DTX or PEG-HMS-DTX was dissolved in 10 mL of PBS (pH 7.4); for the reductive condition, 10 mM glutathione (GSH) was added to the buffer. Each dispersion was loaded into a dialysis membrane (molecular-weight cut-off ≈ 3500 Da) and submerged in 100 mL of PBS (pH 7.4) incubated at 37 °C with gentle shaking (≈ 70 rpm) in the dark. At designated intervals, an aliquot (2 mL) of the release medium was taken out and substituted with an equal amount of fresh buffer. Released DTX was extracted from the collected medium by liquid–liquid separation using 1 mL of ethyl acetate, and subsequently centrifuged at 5000 rpm for 5 min to remove residual impurities. The organic phase was collected and e*v*aporated under reduced pressure at room temperature, and the dried residue was re-dissolved in acetonitrile for quantitative determination of DTX content *via* HPLC.

### 2.7 Cellular uptake analysis

Cellular internalization of the nanocarriers was assessed by fluorescein isothiocyanate (FITC)-labeled nanoparticles (HMS–FITC and PEG–HMS–FITC). RM-1 cells were cultured in 6-well plates containing sterilized glass coverslips at a seeding amount of 1.5 × 10^5^ cells per well in 2.0 mL of high-glucose DMEM and maintained for 24 h at 37 °C. The culture medium was changed to fresh medium containing free FITC, HMS–FITC, or PEG–HMS–FITC (equivalent FITC concentration: 5.0 μg/mL), and incubation continued for another 4 h to allow nanoparticle internalization. After exposure, cells were gently rinsed several times with PBS (pH 7.4) to eliminate unbound probe, fixed using 4% (*w*/*v*) paraformaldehyde for 20 min at room temperature and later permeabilized and labeled with DAPI (3 min) to visualize nuclei. After washing with PBS, the samples were mounted on glass slides using glycerol mounting solution, and observed under a confocal laser scanning microscope (CLSM, Nikon, Tokyo, Japan) to observe intracellular fluorescence distribution. For quantitative analysis by flow cytometry, RM-1 cells were plated into 6-well plates (2.0 × 10^5^ cells per well) and cultured for 24 h for attachment. The culture medium was substituted with that containing free FITC, HMS–FITC, or PEG–HMS–FITC (FITC 5.0 μg/mL). After incubation for 4 h, cells were rinsed three times with cold PBS to remo*v*e non-internalized nanoparticles, detached using trypsin without EDTA, and redistributed in PBS. Intracellular fluorescence intensity was quantified using a BD FACSCalibur flow cytometer (BD Biosciences, USA) provided with a 488 nm excitation laser. For each sample, no fewer than 10,000 events were recorded and processed by FlowJo software (Tree Star, USA).

### 2.8 Cell viability studies

The cytotoxic effect of the nanocarriers was determined by the MTT colorimetric assay. RM-1 cells were cultured in 96-well plates (8.0 × 10^3^ cells/well, 200 μL high-glucose DMEM) and incubated for 24 h at 37 °C to allow adhesion. After cell adhesion, the medium was substituted with fresh culture medium containing either free docetaxel (DTX) or DTX-loaded nanoparticles at final concentrations varying from 0.31 to 10.0 μg·mL^−1^. Free DTX was dissolved in culture medium containing DMSO, with the final DMSO concentration maintained below 0.1% (*v*/v) to avoid solvent-induced cytotoxicity. Untreated wells served as the control group, and each concentration was tested in triplicate. Following another 24 h of exposure, 20 μL of MTT reagent (5.0 mg·mL^−1^) was introduced into each well, and incubation continued for an additional 4 h to permit formazan crystal generation. The supernatant was aspirated, and 150 μL of dimethyl sulfoxide (DMSO) was used to dissolve the resulting crystals. The absorbance of each well was measured at 490 nm using a microplate spectrophotometer. To evaluate carrier-related cytotoxicity, RM-1 cells were also treated with blank nanoparticles (without DTX) at concentrations of 30–150 μg·mL^−1^ under the same conditions. Cell viability (%) was determined by the following formula:

(3) $$\text{Cell Viability}\;\left(\%\right)=\frac{A\text{sample}}{A\text{control}}\times 100\%$$

### 2.9 Pharmacokinetic study and *in vivo* drug tumor distribution

All animal experiments were conducted in accordance with the ARRIVE guidelines and approved by the Animal Ethics Committee of the First Hospital of Jilin University (Approval No. (2022) JLUH-AEC-0125. All efforts were made to minimize animal suffering and to reduce the number of animals used. Male Sprague–Dawley rats (200–220 g) were employed for pharmacokinetic assessment and randomly divided into three groups (*n* = 3). Each group were administered intravenously with free docetaxel (DTX), HMS–DTX, or PEG–HMS–DTX formulations *via* the tail vein with a dosage equivalent to 5.0 mg·kg^−1^ of DTX. At designated sampling intervals (0.25, 0.5, 1, 2, 4, 8, 12, and 24 h post-injection), blood was drawn from the retro-orbital venous plexus into heparinized microtubes. Plasma was isolated *via* centrifugation at 12,000 rpm for 10 min and stored at −20 °C pending quantification. To precipitate plasma proteins, methanol was added to the samples, and the resulting supernatant was analyzed using high-performance liquid chromatography (HPLC, Agilent Technologies, USA) fitted with a C18 reversed-phase column and ultraviolet detection at 230 nm. Pharmacokinetic indicators, such as the area under the plasma concentration–time curve (AUC₀₋ₜ), half-life (t₁/₂), systemic clearance (CL), and maximum plasma concentration (Cₘₐₓ), were determined through non-compartmental analysis using DAS 3.2 software.

To evaluate the intratumoral accumulation of PEG–HMS–DTX, a subcutaneous prostate tumor model was constructed using male C57BL/6 mice (18–20 g, Animal Experiment Center of Jilin University). Briefly, RM-1 cells (5.0 × 10^5^ cells in 0.1 mL PBS) were inoculated into the right axillary region. When the tumor volume reached approximately 200 mm^3^, the animals were randomly assigned to three groups (*n* = 3) and treated by intra*v*enous injection of free DTX, HMS–DTX, or PEG–HMS–DTX at an equivalent DTX dose of 5.0 mg·kg^−1^. At specific time points (4, 12, and 24 h post-injection), the mice were sacrificed, and tumor tissues were excised for quantitative analysis of DTX distribution.

For tissue extraction, 500 mg of tumor sample was mixed with 1 mL acetonitrile and homogenized under ice-cold conditions. The homogenate was centrifuged at 4000 rpm for 10 min, and the supernatant was collected. A 500 μL aliquot of the extract was combined with 100 μL of an internal standard solution (DTX, 2000 ng·mL^−1^), vortexed for 3 min, and centrifuged again at 13,000 rpm for 5 min. The resulting supernatant was evaporated at 30 °C under nitrogen, reconstituted in 100 μL of acetonitrile, and subjected to HPLC–MS/MS analysis. Chromatographic separation was achieved using a Kinetex XB-C18 reversed-phase column (2.6 μm, 50 × 2.1 mm) with a mobile phase of acetonitrile/water (80:20, *v*/v) containing 0.1% formic acid, operated at a flow rate of 0.2 mL·min^−1^ and a column temperature of 30 °C. Mass spectrometric detection was performed with an electrospray ionization (ESI) source in positive ion mode. The MS/MS parameters were as follows: capillary voltage 3.45 k*V*, cone voltage 64 V, collision energy 64 eV, and desolvation gas flow rate 650 L·h^−1^.

### 2.10 *In vivo* antitumor activity and biosafety evaluation

The therapeutic performance and systemic safety of the nanocarriers were assessed using a subcutaneous RM-1 prostate tumor model constructed in 11-week-old male C57BL/6 mice. In brief, RM-1 cells (5.0 × 10^5^ in 0.1 mL PBS) were implanted into the right axillary region. When tumors reached an approximate *v*olume of 100 mm^3^, the animals were randomly assigned to four groups (*n* = 8): blank control, free DTX, HMS–DTX, and PEG–HMS–DTX. Each group was intravenously injected with the corresponding formulation (DTX dose: 5.0 mg·kg^−1^) in 0.2 mL PBS, whereas the control group was given the same volume of PBS. Drug administration was initiated on day 1 and performed once every 3 days, with a total of four treatments completed by day 17. Tumor size and body weight were monitored every other day throughout the experimental period. After the final treatment, mice were euthanized, and tumors together with major organs (heart, liver, spleen, lung, and kidney) were collected, rinsed thoroughly with PBS, and fixed in 4% (*w*/*v*) paraformaldehyde for histological evaluation. Tumor growth inhibition was analyzed using standard volumetric calculations. Tumor volume (*V*, mm^3^) was determined using the following equation:

(4) $$V\;\left(\mathrm{mm}3\right)=\frac{L\times S2}{2}$$

where *L* and *S* denote the longest and shortest tumor diameters, respectively. The tumor growth inhibition was computed as:

(5) $$\text{Tumor inhibition rate}\;\left(\%\right)=\frac{V_{\text{control}}-V_{\text{sample}}}{V\;\text{control}}\times 100$$

### 2.11 *In vivo* survival analysis of RM-1 tumor-bearing mice

To assess the survival benefits of the formulations, an RM-1 prostate tumor model was constructed using male C57BL/6 mice (6–8 weeks old). Briefly, RM-1 cells (1 × 10^6^) suspended in 0.1 mL of PBS were injected subcutaneously into the right side of the mouse. When the tumors reached a volume of around 100 mm^3^, the animals were randomly assigned to four treatment groups (*n* = 8 per group): saline control, free DTX (5 mg·kg^−1^), HMS–DTX, and PEG–HMS–DTX. Each formulation was deli*v*ered through tail vein injection at intervals of three days, with a total of four administrations. Throughout the treatment period, the lifespan of the mice was monitored from the initiation of dosing until natural death or humane euthanasia. Survival data were processed using GraphPad Prism (version X, GraphPad Software, USA) to generate Kaplan–Meier plots, and intergroup differences were statistically analyzed using the log-rank (Mantel–Cox) test. All animal studies were reviewed and authorized by the Institutional Animal Care and Use Committee (IACUC) of Jilin University and performed in accordance with NIH guidelines for the welfare and handling of laboratory animals.

### 2.12 Histological and immunohistochemical evaluation

Major organs (heart, liver, spleen, lung, and kidney) together with excised tumor tissues were preserved in 4% (*w*/*v*) paraformaldehyde prepared in PBS. After fixation, the samples were dehydrated, embedded in paraffin and sectioned for histological examination. Tissue morphology was examined following hematoxylin–eosin (H&E) staining. To further evaluate apoptosis within tumor regions, immunohistochemical staining was performed using antibodies specific to Caspase-3, Bcl-2, and proliferating cell nuclear antigen (PCNA). Subsequently, fluorescently conjugated secondary antibodies were applied for visualization. The stained sections were visualized using CLSM, and fluorescence signal was semi-quantitatively processed with ImageJ software (NIH, Bethesda, MD, USA).

### 2.13 Elisa and inflammatory cytokine analyses

Serum biochemical parameters were assessed using commercial ELISA kits. Cardiac integrity was examined through the determination of creatine kinase (CK) and its myocardial isoenzyme (CK-MB), while hepatic activity was reflected by alanine aminotransferase (ALT) and aspartate aminotransferase (AST) levels. Renal performance was evaluated *via* measurements of blood urea nitrogen (BUN) and serum creatinine (Cr). All assays were performed in strict compliance with the manufacturer's instructions, with each sample analyzed in triplicate to ensure data reproducibility and accuracy.

To further assess systemic inflammatory reactions elicited by different treatments, blood was collected from RM-1 tumor-bearing mice at the conclusion of the therapeutic course. Following spinning at 3000 rpm for 10 min, serum samples were harvested and preserved at −80 °C until testing. The concentrations of interleukin-6 (IL-6), tumor necrosis factor-α (TNF-α), and interleukin-1β (IL-1β) were measured using ELISA kits. Optical absorbance at 450 nm was recorded with a microplate reader, and cytokine levels were determined based on standard calibration curves.

### 2.14 Statistical analysis

Quantitative data were expressed as the mean accompanied by standard deviation (mean ± SD). Statistical comparisons between two separate datasets were carried out using an unpaired two-tailed Student's *t*-test, whereas variations among multiple experimental groups were assessed through one-way analysis of variance (ANOVA). The exact *P* values corresponding to each dataset are provided within the Fig. legends. Levels of significance were represented as follows: not significant (n.s.), *P* > 0.05; *P* < 0.05; *P < 0.01; **P < 0.001; and ***P < 0.0001.

## 3 Results and discussion

### 3.1 Comprehensive physicochemical characterization of HMS-based nanoparticles

**Fig. 1:**
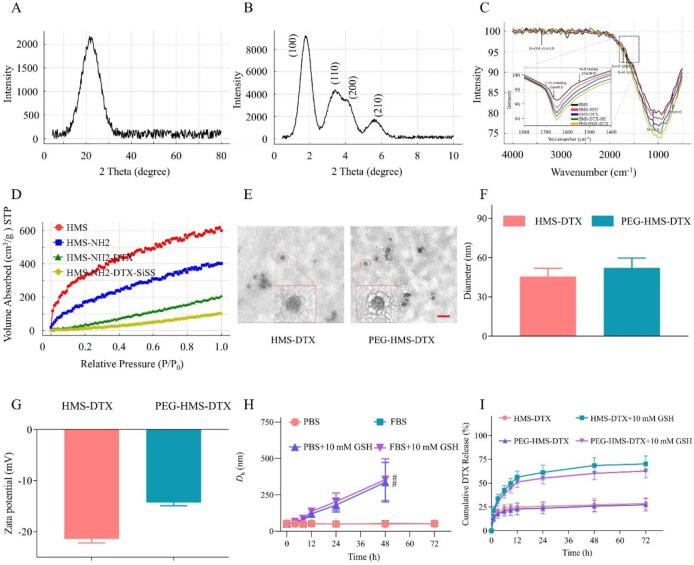
Comprehensive physicochemical characterization of HMS-derived nanocarriers. (A) Wide-angle XRD patterns of HMS. (B) Small-angle XRD profiles of HMS confirming mesostructural ordering. (C) FTIR spectra of HMS, HMS-NH2, HMS-DTX, HMS-DTX-SH, and PEG-HMS-DTX, illustrating the stepwise surface functionalization of HMS-based nanocarriers. (D) Nitrogen adsorption–desorption isotherms of HMS, HMS–NH₂, HMS–NH₂–DTX, and HMS–NH₂–DTX–SiSS revealing porosity changes. (E) Representative TEM images of HMS-DTX and PEG-HMS-DTX nanoparticles at low and high magnifications. (Scale bar = 100 nm). (F) Hydrodynamic size distributions of HMS–DTX and PEG–HMS–DTX obtained by DLS. (G) Zeta potential analysis of HMS–DTX and PEG–HMS–DTX. (H) Temporal particle size evolution of HMS–DTX and PEG–HMS–DTX in PBS or FBS containing 0 or 10 mM GSH. (I) Cumulative DTX release behavior of HMS–DTX and PEG–HMS–DTX in PBS with or without 10 mM GSH at 37 °C.

**Scheme 1:**
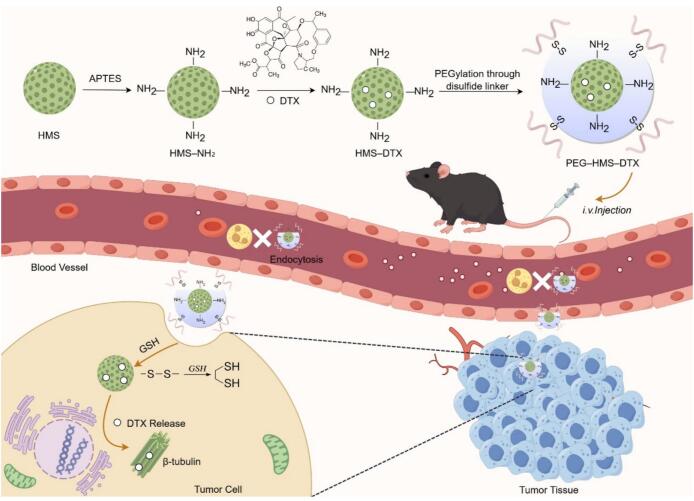
Schematic representation of PEG–HMS–DTX fabrication and its antitumor action mechanism. The nanocarrier was prepared by introducing disulfide-bonded PEG onto DTX-encapsulated HMS. Following intravenous administration, the nanoparticles preferentially accumulate within tumor sites through the EPR effect, where intracellular GSH induces PEG cleavage and stimulates DTX release, subsequently promoting microtubule stabilization and apoptotic cell death.

The wide-angle XRD pattern (Fig. 1A) exhibited a broad diffraction peak centered near 22°, typical of amorphous silica frameworks, indicating that all HMS-based nanoparticles maintained a disordered mesoporous structure without long-range crystallinity. The small-angle XRD curve (Fig. 1B) revealed a distinct reflection near 2–3°, corresponding to the (100) plane of the ordered mesostructure, verifying the successful construction of hexagonally arranged mesopores within the silica matrix. Progressive attenuation of the diffraction peak intensity after amination and drug loading suggested partial pore filling and framework modification. As shown in Fig. 1C, Fourier transform infrared (FTIR) spectra further confirmed the stepwise functionalization of the HMS-based nanoplatform. The characteristic Si–O–Si stretching vibrations were observed at approximately 1080 and 800 cm^−1^, accompanied by a Si–OH bending band around 950 cm^−1^. After amine functionalization (HMS–NH_2_), new absorption features appeared at ∼1560 and 1470 cm^−1^, which can be attributed to N—H bending and C—N stretching vibrations, respectively. Upon DTX loading, additional bands emerged at around 1730 cm^−1^ (C=O stretching) and 1240 cm^−1^ (C–O–C vibration), indicating the successful incorporation of DTX into the nanocarrier. Following PEG grafting, a new absorption band near 2880 cm^−1^ corresponding to C—H stretching of the PEG backbone was observed, together with an intensified and broadened band around 1100 cm^−1^ associated with C–O–C vibrations, supporting the successful PEGylation of the nanoparticles. Nitrogen adsorption–desorption measurements (Fig. 1D) exhibited typical type IV isotherms with pronounced hysteresis loops, characteristic of mesoporous materials. The BET surface area of pristine HMS reached 865.7 m^2^ g^−1^ and decreased sequentially to 598.2, 475.3, and 322.6 m^2^ g^−1^ for HMS–NH₂, HMS–NH₂–DTX, and HMS–NH₂–DTX–SiSS, respectively, owing to stepwise surface modification and drug occupation within the pores. Similarly, pore volume and average pore diameter decreased slightly, further confirming successful DTX loading and disulfide crosslinking. These results collectively validate that the mesoporous silica architecture remained structurally intact after sequential amine functionalization, disulfide introduction, and PEGylation. The observed changes in surface chemistry and porosity demonstrate that each synthetic step effectively contributed to the intended chemical architecture — an internally crosslinked, redox-responsive nanocarrier with abundant mesopores for drug accommodation. Such structural integrity and functional tunability are essential to achieving controlled DTX release and tumor-specific responsiveness in subsequent experiments. (See Scheme 1.)

Transmission electron microscopy (TEM) revealed that both HMS-DTX and PEG-HMS-DTX nanoparticles exhibited uniform and spherical morphologies with good structural integrity (Fig. 1E). The enlarged views further highlight the consistent particle shape and internal contrast variations of individual nanoparticles, suggesting that drug loading and subsequent surface modification did not induce obvious structural collapse. It should be noted that quantitative pore size analysis was not conducted in this study, as nitrogen adsorption measurements may be compromised by pore blocking effects after drug loading and surface modification. Dynamic light scattering (DLS) analysis demonstrated that the HMS-DTX nanoparticles possessed a mean particle diameter of approximately 50 nm, with a low polydispersity index (PDI = 0.22 ± 0.03), indicating a narrow size distribution. After PEGylation, PEG-HMS-DTX exhibited a slightly increased hydrodynamic diameter along with a similarly low PDI value (0.17 ± 0.02), confirming good colloidal homogeneity (Fig. 1F). After PEGylation, PEG-HMS-DTX exhibited a moderately negative zeta potential of approximately −15 mV, which is consistent with partial surface shielding by PEG chains rather than complete charge neutralization (Fig. 1G). PEG-HMS-DTX exhibited excellent colloidal stability in both PBS and serum-containing media (FBS) for at least 72 h (Fig. 1H), indicating that PEGylation effectively stabilizes the nanoparticles under physiologically relevant conditions. In contrast, exposure to a reductive environment containing glutathione (GSH) induced pronounced changes in hydrodynamic size and dispersity. This behavior is likely associated with the reduction of disulfide linkages, which leads to structural relaxation of the surface architecture and increased hydration of the nanoparticle interface. Such redox-triggered size expansion reflects alterations in the interfacial layer rather than particle aggregation, a phenomenon that has been widely reported in disulfide-bridged nanocarrier systems. Collectively, these results demonstrate that the PEGylated, disulfide-bridged mesoporous silica carrier remains stable under physiological conditions while undergoing responsive structural changes in reductive environments.

Mechanistically, the observed physicochemical profile explains several downstream behaviors. The presence of a PEG corona offers steric hindrance that minimizes nonspecific protein binding and opsonization, which in turn extends systemic circulation and enhances passive tumor targeting through the EPR effect; the shift of ζ-potential toward neutrality further mitigates nonspecific electrostatic interactions with serum proteins and endothelial surfaces. The internal/disulfide bridging within the silica matrix supplies an intrinsic, microenvironment-sensitive trigger: under tumor-relevant reductive conditions (elevated intracellular GSH), disulfide cleavage weakens the framework/PEG linkage and facilitates payload release or particle disassembly. This “stable in blood — trigger in tumor” behavior is precisely the design goal for improving therapeutic index by minimizing premature leakage while enabling efficient intratumoral drug liberation.

The *in vitro* release study demonstrated that PEG-HMS-DTX maintained good stability under physiological conditions (PBS, pH 7.4) with less than 30% cumulative drug release, whereas the appearance of 10 mM GSH markedly accelerated drug liberation, reaching approximately 60% release after 72 h (Fig. 1I). HMS-DTX showed a comparatively less controlled release, underscoring the critical role of PEGylation and disulfide linkages in regulating drug kinetics. This redox-triggered release is highly relevant to the tumor microenvironment (TME), where intracellular GSH concentrations are significantly elevated compared with blood plasma (Chen et al., 2013; Cheng et al., 2011). The selective release profile therefore reduces systemic drug exposure while maximizing intratumoral delivery. Similar redox-sensitive HMS systems have been reported to improve chemotherapy efficacy, but the present design integrates both internal disulfide bridging and external PEGylation, thereby enhancing both stability and responsiveness—a key advancement over earlier single-trigger nanocarriers.

### 3.2 Cellular internalization and *in vitro* antitumor activity

**Fig. 2:**
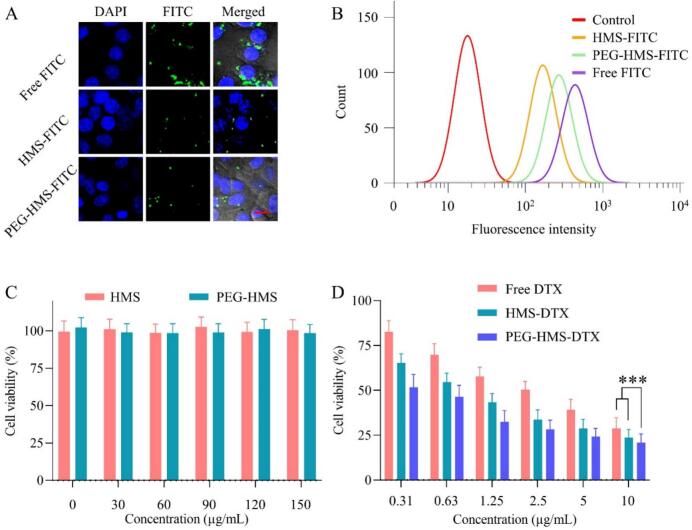
Evaluation of cellular uptake and *in vitro* cytotoxicity of HMS-derived nanoparticles. (A) CLSM micrographs of RM-1 cells after incubation with free FITC, HMS–FITC, and PEG–HMS–FITC for 4 h. The nuclei were visualized using DAPI staining (blue), and FITC fluorescence was detected in green; scale bar = 20 μm. (B) Flow cytometry analysis displaying fluorescence intensity profiles of RM-1 cells treated with control, free FITC, HMS–FITC, or PEG–HMS–FITC. (C) Viability of RM-1 cells exposed to increasing concentrations of HMS and PEG–HMS for 24 h. (D) Cytotoxicity of free DTX, HMS–DTX, and PEG–HMS–DTX against RM-1 cells at different concentrations after 24 h incubation (***P < 0.001). (For interpretation of the references to colour in this figure legend, the reader is referred to the web version of this article.)

Confocal fluorescence microscopy revealed markedly stronger nanoparticle-associated fluorescence in RM-1 cells treated with PEG-HMS-FITC compared with HMS-FITC (Fig. 2A). Similarly, PEG-HMS-DTX exhibited enhanced cellular association relative to non-PEGylated formulations, as evidenced by increased intracellular fluorescence signals distributed throughout the cell region. These observations indicate that PEGylation did not impair nanoparticle–cell interactions. Instead, the PEG coating provided colloidal stabilization while maintaining effective cellular association, consistent with previous reports on appropriately designed PEGylated nanocarriers (Chuang et al., 2010). Such a balance is particularly important, since excessive PEG shielding may mask binding sites and impair uptake, while insufficient PEGylation compromises circulation stability. Our findings suggest that PEG-HMS-DTX achieves an optimal trade-off between stealth properties and cellular entry. Quantitative analysis of intracellular fluorescence intensity further supported enhanced cellular association of PEG-HMS-DTX compared with non-PEGylated formulations (Fig. 2B). This analysis reflects relative differences in nanoparticle–cell interactions rather than the absolute percentage of nanoparticle-positive cells. As shown in Fig. 2B, flow cytometric results demonstrated distinct fluorescence intensity shifts among the three groups. PEG–HMS–FITC exhibited markedly stronger intracellular fluorescence compared with HMS–FITC, indicating that surface PEG modification promoted nanoparticle internalization, likely through improved colloidal stability and reduced aggregation. Quantitative fluorescence analysis aligned well with CLSM imaging results (Fig. 2A), confirming efficient endocytic uptake of FITC-tagged nanoparticles by tumor cells. Such controlled cellular uptake behavior is beneficial for *in vivo* applications, as PEG modification can balance the trade-off between prolonged systemic circulation and sufficient tumor cell internalization, thus optimizing the nanoparticle delivery efficiency for subsequent therapeutic evaluations.

Cell viability assays provided further insights into therapeutic potential. As shown in Fig. 2C, empty HMS and PEG-HMS nanoparticles exhibited negligible cytotoxicity across a broad concentration range, confirming the excellent biocompatibility of the mesoporous silica framework. In contrast, DTX-loaded formulations induced pronounced dose-dependent cytotoxicity against RM-1 cells (Fig. 2D). Among them, PEG-HMS-DTX exhibited enhanced inhibitory effects compared with free DTX and HMS-DTX, particularly at higher drug concentrations, where statistically significant differences were observed. Based on the dose–response curves, the IC_50_ values were estimated to be approximately 2.6 μg/mL for free DTX, 0.9 μg/mL for HMS-DTX, and 0.4 μg/mL for PEG-HMS-DTX. Among the tested formulations, PEG-HMS-DTX exhibited the lowest IC_50_ value, indicating enhanced cytotoxic potency. Although PEGylation is often associated with reduced nonspecific cellular interactions, PEG-HMS-DTX exhibited higher cytotoxicity than HMS-DTX. This behavior can be attributed to the improved colloidal stability imparted by the PEG corona, which prevents nanoparticle aggregation in culture medium and maintains a higher effective concentration of dispersed particles at the cell surface (Wen et al., 2012). Consequently, PEG-HMS-DTX achieves enhanced cellular internalization and, upon intracellular exposure to elevated glutathione levels, undergoes disulfide cleavage to promote efficient docetaxel release. This combination of stability-driven uptake and redox-responsive intracellular drug liberation results in amplified cytotoxicity compared with non-PEGylated counterparts. These results collectively highlight the dual advantages of the PEG-HMS-DTX system: robust cellular uptake and redox-responsive intracellular drug release. Compared with free DTX, which diffuses nonspecifically and is quickly metabolized, PEG-HMS-DTX maintains a controlled and localized release profile, thereby increasing therapeutic efficacy while reducing systemic toxicity. Similar redox-sensitive nanocarriers have been reported in recent studies, but our system advances this concept by integrating PEGylation without compromising uptake efficiency—a limitation in many earlier designs. In summary, the cellular uptake and cytotoxicity results establish a strong mechanistic basis for the improved *in vivo* outcomes observed in later experiments. The dual capability of PEG-HMS-DTX to penetrate tumor cells effectively and respond to intracellular redox cues positions it as a promising next-generation nanomedicine for prostate cancer management.

### 3.3 Pharmacokinetic behavior and *in vivo* tumor distribution

**Fig. 3:**
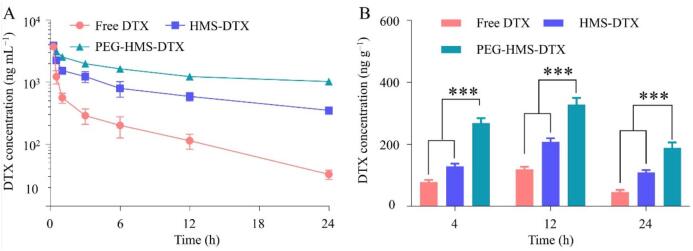
*In vivo* pharmacokinetic behavior and biodistribution of DTX-loaded formulations. (A) Time-dependent plasma concentration curves of free DTX, HMS–DTX, and PEG–HMS–DTX following intravenous injection in rats (DTX dose: 5.0 mg kg^−1^). (B) Quantitative determination of DTX levels in tumor tissues at 4, 12, and 24 h after administration of various formulations (***P < 0.001).

It should be noted that the stability evaluated *in vitro* (Fig. 1H and I) reflects short-term colloidal integrity in simplified media, whereas the pharmacokinetic profiles in Fig. 3 represent systemic stability *in vivo* under continuous circulation and biological clearance processes. Although both HMS-DTX and PEG-HMS-DTX exhibit comparable colloidal stability *in vitro*, PEGylation confers a distinct advantage *in vivo* by reducing protein adsorption and reticuloendothelial uptake, thereby prolonging blood circulation. As illustrated in Fig. 3A, distinct pharmacokinetic behaviors were observed among the three tested formulations. Free DTX displayed a rapid decline in plasma concentration, indicating fast systemic clearance and limited circulation persistence. HMS–DTX moderately extended the drug circulation period, reflecting the stabilizing effect of mesoporous encapsulation. In contrast, PEG–HMS–DTX exhibited a significantly prolonged blood retention, maintaining higher plasma levels throughout the 24 h observation period. Quantitatively, compared with free DTX, PEG–HMS–DTX showed approximately 2.3- and 2.1-fold elevations in AUC₀–t and t₁/₂ values, respectively, while the clearance rate was substantially reduced. These results confirm that PEG modification effectively minimizes opsonization and reticuloendothelial clearance, leading to sustained systemic exposure. The enhanced pharmacokinetic behavior of PEG–HMS–DTX can be attributed to two key factors. First, the PEG corona forms a steric and hydrated barrier that reduces nonspecific plasma protein adsorption and subsequent opsonization, thereby minimizing macrophage uptake and prolonging blood circulation, as widely reported for PEGylated nanocarriers (Gref et al., 1994; Owens III and Peppas, 2006). Second, the mesoporous framework enables diffusion-controlled drug release, contributing to a more sustained systemic exposure. Collectively, these features provide a mechanistic basis for the improved pharmacokinetic profile of PEG–HMS–DTX and support its enhanced tumor accumulation and therapeutic performance observed in subsequent experiments.

Biodistribution profiling directly demonstrated the delivery performance of various formulations in RM-1 tumor–bearing mice. As depicted in Fig. 3B, free DTX underwent rapid systemic clearance, leading to minimal drug accumulation in tumors at 4, 12, and 24 h post-administration. HMS-DTX exhibited moderately improved tumor accumulation, reflecting the stabilizing effect of mesoporous encapsulation. In contrast, PEG-HMS-DTX achieved significantly higher intratumoral drug levels throughout all time intervals, with a marked peak at 12 h post-injection, demonstrating its superior tumor-targeting capability and sustained retention. This improved tumor distribution is the result of several synergistic factors. PEG modification minimized nonspecific protein adsorption and hindered interaction with the reticuloendothelial system, resulting in extended blood circulation. In addition, the optimized nanoscale dimension (approximately 40–50 nm) facilitated passive tumor accumulation *via* the EPR effect, as particles of this size preferentially extravasate through the leaky tumor vasculature. Moreover, the disulfide-bridged framework enabled redox-sensitive drug release, ensuring that DTX remained protected during circulation but was selectively liberated within the reductive tumor microenvironment. Together, these features not only enhanced tumor enrichment but also ensured that the drug was released precisely where therapeutic action was required. Compared with free DTX, which distributes broadly and causes significant systemic toxicity, PEG-HMS-DTX markedly increased local tumor exposure while reducing off-target distribution to normal organs. This selective accumulation is directly correlated with the enhanced therapeutic efficacy observed in subsequent antitumor experiments. These findings are consistent with earlier studies on PEG-modified silica nanocarriers, which also reported prolonged circulation and improved tumor accumulation. However, the present system advances this concept by incorporating internal disulfide linkages, which provide an additional layer of tumor-specific responsiveness, further improving delivery precision. Collectively, the biodistribution results highlight the pharmacokinetic advantages of PEG-HMS-DTX, establishing a mechanistic basis for its superior therapeutic performance. By integrating long circulation, EPR-mediated tumor accumulation, and tumor-triggered drug release, this nanoplatform achieves a favorable balance between efficacy and safety, offering a clear translational advantage over conventional docetaxel formulations.

### 3.4 Antitumor efficacy and apoptotic mechanisms

**Fig. 4:**
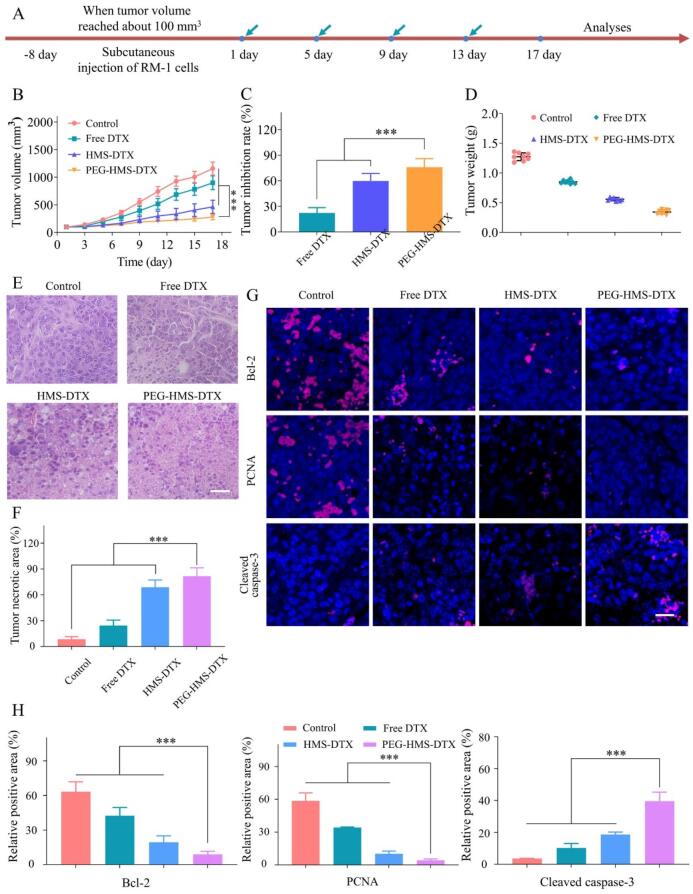
*In vivo* therapeutic performance and histopathological assessment of DTX-loaded nanoformulations. (A) Experimental schedule for the treatment of RM-1 tumor-bearing mice. (B) Tumor growth profiles of mice receiving saline, free DTX, HMS–DTX, or PEG–HMS–DTX during the 17-day therapy period. (C) Statistical evaluation of tumor inhibition rates across treatment groups (***P < 0.001). (D) Final tumor weights of mice following 17 days of administration. (E) Representative H&E micrographs of tumor sections from various groups (scale bar = 200 μm). (F) Quantitative evaluation of necrotic regions within tumor tissues (***P < 0.001). (G) Immunofluorescence images showing Bcl-2, PCNA, and cleaved caspase-3 expression in tumor tissues from different formulations (scale bar = 100 μm). (H) Quantification of positive staining areas for Bcl-2, PCNA, and cleaved caspase-3 (***P < 0.001).

The therapeutic efficacy of various formulations was comprehensively assessed in RM-1 tumor–bearing mice (Fig. 4A). As illustrated in Fig. 4B, the control group displayed fast and continuous tumor growth, with volumes surpassing ∼1200 mm^3^ by day 17. Free DTX moderately delayed tumor progression, but tumor growth remained significant, reflecting the limitations of conventional chemotherapy such as rapid clearance and insufficient tumor accumulation. HMS-DTX further improved tumor inhibition, indicating that mesoporous encapsulation could prolong drug retention and enhance therapeutic outcomes. Notably, PEG–HMS–DTX showed the strongest therapeutic performance, keeping tumor volumes below 300 mm^3^ during the entire treatment and yielding a tumor inhibition rate exceeding 75%, outperforming HMS–DTX (∼60%) and free DTX (∼22%) (Fig. 4C). Tumor weights at sacrifice confirmed this trend (Fig. 4D). Histological and immunohistochemical analyses further elucidated the mechanisms underlying these therapeutic outcomes. H&E staining revealed extensive necrotic regions in tumors from PEG-HMS-DTX–treated mice, while necrosis was limited in the HMS-DTX and free DTX groups (Fig. 4E). Semi-quantitative analysis confirmed that PEG-HMS-DTX induced the largest necrotic tumor fraction (Fig. 4F). At the molecular level, immunohistochemistry showed a significant decrease in proliferative marker PCNA and anti-apoptotic protein Bcl-2, along with a marked increase in cleaved caspase-3 expression (Fig. 4G). Quantitative image analysis validated these changes, indicating that PEG-HMS-DTX effectively inhibited proliferation while simultaneously activating apoptosis pathways (Fig. 4H).

Collectively, the data indicate that the remarkable therapeutic performance of PEG–HMS–DTX results from synergistic effects of improved tumor localization, reduction-responsive drug release, and apoptosis induction. The reduction of Bcl-2 expression alongside caspase-3 activation implies involvement of the mitochondrial apoptotic cascade, which is a critical mechanism for overcoming drug resistance in prostate cancer. Previous studies have reported that conventional DTX often fails to induce sufficient apoptosis due to inadequate intracellular delivery,(Wang et al., 2011) whereas PEG-HMS-DTX achieves higher intratumoral concentration and controlled release, thereby potentiating downstream apoptotic signaling.

From a translational perspective, the ability of PEG-HMS-DTX to simultaneously suppress proliferation and induce apoptosis provides a dual mechanism of tumor regression that directly addresses the clinical limitations of DTX monotherapy. The enhanced therapeutic index demonstrated here underscores the potential of PEG-HMS-DTX as a next-generation nanomedicine platform, with the capability to achieve robust tumor suppression while minimizing systemic side effects. This mechanistic insight not only validates the design rationale but also provides strong preclinical evidence supporting further development of PEG-HMS-DTX toward clinical application in prostate cancer therapy.

### 3.5 *In vivo* biosafety assessment

**Fig. 5:**
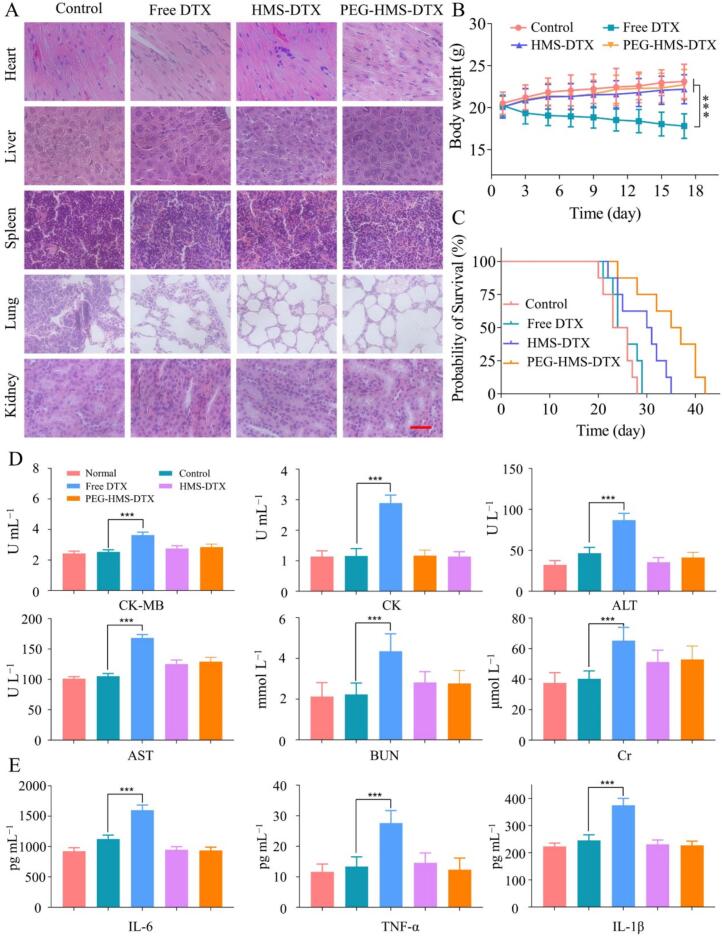
*In vivo* biosafety and systemic toxicity assessment of DTX-based nanoformulations. (A) Representative H&E micrographs of major organs, obtained from mice treated with saline, free DTX, HMS–DTX, or PEG–HMS–DTX (scale bar = 500 μm). (B) Body weight fluctuations in tumor-bearing mice over 17 days following administration of different formulations (***P < 0.001). (C) Kaplan–Meier survival analysis of mice from each group during the 42-day observation period. (D) Serum biochemical parameters—CK-MB, CK, ALT, AST, BUN, and Cr—measured to evaluate hepatic, renal, and cardiac functions after treatment (***P < 0.001). (E) Quantitative comparison of serum inflammatory cytokines (IL-6, TNF-α, and IL-1β) among treatment groups (***P < 0.001).

The systemic biosafety of PEG–HMS–DTX was assessed by combining histopathological examination and serum biochemical analysis. As illustrated in Fig. 5A, H&E staining of key organs (heart, liver, spleen, lung, and kidney) demonstrated well-preserved tissue morphology without observable necrosis, inflammation, or structural damage in the PEG–HMS–DTX-treated mice. In contrast, free DTX–treated mice displayed mild hepatic vacuolar degeneration and focal renal tubular changes, consistent with the well-documented hepatotoxicity and nephrotoxicity of conventional DTX. HMS-DTX slightly alleviated these toxic effects, but occasional tissue alterations were still observed, underscoring the added benefit of PEGylation. Notably, mice treated with PEG–HMS–DTX maintained stable body weights throughout the study, indicating negligible systemic toxicity (Fig. 5B), suggesting enhanced efficacy was not accompanied by systemic toxicity. Serum biochemical analyses further confirmed these observations (Fig. 5D). In the PEG–HMS–DTX–treated mice, cardiac markers (CK, CK-MB), hepatic enzymes (ALT, AST), and renal indicators (BUN, Cr) all stayed within physiological ranges, showing no significant differences from the control group. In contrast, free DTX induced significant elevations in ALT, AST, BUN, and Cr, reflecting hepatic and renal stress, while HMS-DTX showed partial improvement but not complete normalization. These findings indicate that PEG-HMS-DTX effectively reduces systemic toxicity while preserving therapeutic potency. As shown in Fig. 5E, serum cytokine measurements revealed marked differences in inflammatory response among the groups. As opposed to the control group, mice receiving free DTX showed markedly increased serum cytokine concentrations (IL-6, TNF-α, and IL-1β), suggesting that systemic exposure to DTX triggered acute inflammatory responses. In contrast, the HMS-DTX group showed moderate cytokine levels, while PEG-HMS-DTX treatment maintained cytokine concentrations close to the baseline, suggesting excellent systemic biocompatibility and minimal inflammatory stimulation.

The improved biosafety profile can be explained by several design features. PEGylation enhanced colloidal stability and reduced nonspecific protein adsorption, limiting recognition and removal by the reticuloendothelial system, and consequently decreasing drug accumulation in healthy organs. The disulfide-bridged framework ensured that DTX release was largely restricted to the reductive tumor microenvironment, preventing premature leakage during circulation. In addition, the higher tumor accumulation observed in biodistribution studies directly contributed to reduced systemic exposure, thereby sparing non-target tissues from drug-induced injury. These results are consistent with prior reports demonstrating that PEG-modified mesoporous silica nanoparticles reduce off-target toxicity compared with free chemotherapeutics. However, the current system further advances this concept by integrating internal disulfide linkages, which add an additional layer of tumor-specific responsiveness. Such dual protection—PEG shielding during circulation and disulfide-triggered release in the tumor—provides a strong mechanistic basis for the observed safety improvements.

From a translational perspective, the absence of significant histological damage and the normalization of biochemical markers strongly support the potential of PEG-HMS-DTX as a clinically viable formulation with a favorable therapeutic index. Nevertheless, comprehensive long-term toxicity and immunogenicity studies, as well as GLP-compliant pharmacokinetic evaluations, will be necessary to fully establish its safety profile and accelerate its clinical development.

### 3.6 *In vivo* survival analysis

As shown in Fig. 5C, Kaplan–Meier survival curves demonstrated significant variations in overall survival among the different treatment groups. Mice administered free DTX exhibited rapid mortality within 28 days, which was accompanied by significant body weight loss (Fig. 5B), suggesting severe systemic toxicity and limited therapeutic benefit. In contrast, those receiving HMS–DTX or PEG–HMS–DTX showed a pronounced improvement in survival rate, with median survival times extended to approximately 32 and 36 days, respectively (*P* < 0.01 *vs.* free DTX). The prolonged survival observed in the PEG–HMS–DTX–treated mice was correlated with steady body weight and normal histological features of major organs, as shown by H&E staining (Fig. 5A), demonstrating the low systemic toxicity of the PEGylated nanoparticles. These findings highlight that the combination of a mesoporous silica framework with PEG surface modification effectively enhances the biosafety and pharmacological persistence of docetaxel-loaded nanocarriers. The PEGylation improved colloidal stability and minimized opsonization, thereby prolonging systemic circulation and facilitating preferential tumor accumulation. Concurrently, the disulfide-responsive linker structure in the carrier enabled controlled DTX release in the reductive tumor microenvironment, ensuring effective drug exposure at the tumor site while mitigating off-target toxicity. Compared with free DTX, which displayed rapid clearance and nonspecific tissue damage, PEG–HMS–DTX achieved a favorable therapeutic balance between efficacy and safety. The extended survival duration further supports that rational surface modification and redox-sensitive nanocarrier design can significantly improve DTX chemotherapy outcomes in prostate cancer models.

Taken together, the above results highlight the advantages of integrating redox responsiveness with surface-engineered stability in mesoporous silica-based nanocarriers. GSH-responsive mesoporous silica nanoparticles have been extensively investigated as intracellular drug delivery platforms, exploiting elevated reductive conditions in tumor cells to trigger payload release (He and Shi, 2011; Vallet-Regí et al., 2017). Previous studies have demonstrated that disulfide-bridged MSN systems can effectively enhance intracellular drug release and antitumor efficacy;(Lu et al., 2010; Meng et al., 2010) however, many of these systems suffer from limited colloidal stability, premature clearance, or compromised pharmacokinetic performance due to insufficient surface protection (He et al., 2011; Huang et al., 2010).

PEGylation has been widely adopted to improve the circulation stability of nanocarriers, although excessive PEG shielding may attenuate cellular interactions and reduce therapeutic efficacy (Bertrand et al., 2017; Owens III and Peppas, 2006). In this context, the present PEG–HMS–DTX system integrates a disulfide-bridged mesoporous framework with a carefully designed PEG corona, achieving a balance between systemic stability and intracellular responsiveness. Compared with previously reported GSH-responsive MSN formulations, PEG–HMS–DTX exhibits prolonged blood circulation, enhanced tumor accumulation, and reduced systemic toxicity, while maintaining effective intracellular drug release under reductive conditions (Cheng et al., 2011).

Importantly, rather than relying on complex multicomponent architectures, the current design employs a relatively simple and modular strategy, which may facilitate reproducibility and future translational development. Together, these features distinguish PEG–HMS–DTX from existing GSH-responsive mesoporous nanocarriers and support its potential as a practical platform for prostate cancer chemotherapy.

## 4 Conclusion

In this work, we constructed a PEGylated, disulfide-bridged mesoporous silica nanocarrier (PEG–HMS–DTX) for targeted DTX delivery in prostate cancer treatment. The nanoparticles exhibited uniform morphology, nanoscale dimensions, and excellent colloidal stability, while maintaining a reduction-responsive release pattern that minimized premature leakage and enabled efficient drug liberation within the tumor's reductive microenvironment. *In vitro* studies demonstrated that PEG–HMS–DTX was effectively internalized by RM-1 prostate cancer cells, producing higher cytotoxicity than either free DTX or non-PEGylated formulations. *In vivo* biodistribution further confirmed enhanced tumor accumulation, which was consistent with PEG-mediated prolonged circulation and the EPR effect. Therapeutically, PEG–HMS–DTX displayed superior tumor growth inhibition through apoptosis induction while maintaining favorable systemic safety. Histopathological and biochemical analyses verified the biocompatibility of this platform, showing minimal hepatic and renal toxicity as opposed to free DTX. Overall, this study highlights the advantages of integrating mesoporous structure, PEGylation, and disulfide responsiveness into one multifunctional nanosystem, achieving an optimal balance between circulation stability and redox-triggered release. This smart delivery strategy not only improves antitumor effect but also mitigates systemic side effects, providing a promising avenue for enhanced chemotherapy in prostate cancer management.

## CRediT authorship contribution statement

**Yangyang Song:** Writing – original draft, Data curation. **Xue Tan:** Writing – original draft, Data curation. **Kai Yu:** Methodology, Formal analysis. **Yue Wang:** Visualization, Supervision. **Jixue Wang:** Writing – review & editing, Visualization, Supervision, Funding acquisition.

## Consent for publication

Not applicable.

## Funding

This work was supported by the 10.13039/501100001809National Natural Science Foundation of China (Grant No. 82202879), the Natural Science Foundation Project of Jilin Province (YDZJ202301ZYTS100), and Talent Reserve Program (*TRP*)The First Hospital of Jilin University (JDYY-TRP-2025002).

## Declaration of competing interest

The authors declare no conflict of interest in relation to this study. The research was conducted independently, and no financial or non-financial relationships influenced the results or conclusions of this work.

## Data Availability

Data will be made available on request.
